# PRINS Non-Coding RNA Regulates Nucleic Acid-Induced Innate Immune Responses of Human Keratinocytes

**DOI:** 10.3389/fimmu.2017.01053

**Published:** 2017-08-29

**Authors:** Judit Danis, Anikó Göblös, Zsuzsanna Bata-Csörgő, Lajos Kemény, Márta Széll

**Affiliations:** ^1^Department of Dermatology and Allergology, University of Szeged, Szeged, Hungary; ^2^MTA-SZTE Dermatological Research Group, Szeged, Hungary; ^3^Department of Medical Genetics, University of Szeged, Szeged, Hungary

**Keywords:** PRINS, long non-coding RNA, interleukin-6, CCL-5, keratinocyte, poly(dA:dT)

## Abstract

Cytosolic DNA fragments are recognized as pathogen- and danger-associated molecular patterns that induce a cascade of innate immune responses. Moreover, excessive cytosolic DNA can enhance chronic inflammation, predominantly by activating inflammasomes, and thereby contributing to the pathogenesis of chronic diseases, such as psoriasis. Psoriasis associated non-protein coding RNA induced by stress (PRINS) is a long non-coding RNA, which has been shown to be associated with psoriasis susceptibility and cellular stress responses; however, the precise mechanism of its action has not been determined. Here, we provide evidence that, in addition to inflammasome activation, cytosolic DNA induces intracellular inflammatory reactions while decreasing PRINS gene expression. Furthermore, PRINS overexpression can ameliorate the inflammatory-mediator production of keratinocytes induced by cytosolic DNA. Overexpression of PRINS resulted in decreased interleukin-6 (IL-6) and chemokine (C–C motif) ligand 5 (CCL-5) expression and secretion. *In silico* analysis predicted direct binding sites between PRINS and the mRNAs, which was confirmed by an *in vitro* binding assay and on cellular level. Our results indicated that PRINS binds directly to the mRNAs of IL-6 and CCL-5 at specific binding sites and eventually destabilizes these mRNAs, leading to a decrease in their expression and secretion of the corresponding proteins. These results may indicate a restrictive role for PRINS in inflammatory processes.

## Introduction

The skin provides the first line of defense against a variety of environmental, chemical, and physical stimuli and acts as an active member of the innate immune system. Double-stranded DNA (dsDNA) fragments are known to induce antiviral responses in keratinocytes (1, 2) as well as induce inflammasome activation and subsequent interleukin (IL)-1β release in these cells (3, 4). Moreover, keratinocytes express a wide range of pattern recognition receptors (PRR) for nucleotide fragments (5, 6), mainly implicated in antiviral reactions (2, 6). These PRRs are required for immune response in an acute infection and might also lead to the exacerbation of chronic inflammatory inherited multifactorial diseases, such as psoriasis (7). Psoriasis is caused by the interplay of professional immune cells and keratinocytes. Cytosolic nucleotide fragments are highly abundant in psoriatic skin (3), leading to chronic activation of professional immune cells (8), and are thought to be an initiator factor in the disease. In particular, cytosolic nucleotide fragments do not lead to antiviral reaction, but instead activate the AIM2 inflammasome signaling in psoriatic keratinocytes (3), leading to prolonged inflammation.

In the last decade, genome-scale transcription studies have uncovered non-coding RNAs as previously unrecognized players in the dysregulation of inflammatory reaction in psoriatic skin (9, 10). The contribution of both microRNAs (miRNAs) and long non-coding RNAs (lncRNAs) to psoriasis has been extensively studied (11, 12). We were first to identify a lncRNA potentially contributing to disease susceptibility: PRINS, the psoriasis susceptibility-related non-coding RNA induced by stress, which is highly expressed in psoriatic non-lesional epidermis compared both to lesional or healthy epidermis (13). The expression of PRINS is modified by a diverse set of cellular stressors, including starvation, ultraviolet B (UVB) irradiation, translation inhibition (14), and hypoxia (15). Moreover, microbial stimuli decreased PRINS expression in macrophages (16) and normal human epidermal keratinocytes (NHEKs) (14), and PRINS was recently shown to interact with chemokine (C–C motif) ligand 5 (CCL-5, also known as RANTES) in kidney epithelial cells (15). CCL-5 is a chemokine attracting leukocytes to the site of inflammation. Under inflammatory conditions, keratinocytes produce large amount of CCL-5 to attract antigen-presenting cells (17) and T-cells into the epidermis (18); moreover, CCL-5 is overexpressed in atopic dermatitis and psoriatic lesions (19). Based on these findings, we aimed to investigate whether the PRINS lncRNA contributes to keratinocyte innate immune responses.

According to our results, PRINS can ameliorate dsDNA-induced keratinocyte immune responses. Bioinformatic analysis of the PRINS lncRNA sequence revealed putative interaction sites for interleukin-6 (IL-6) and CCL-5 mRNAs. Destruction of the putative interacting region resulted in the loss of the ability of PRINS to bind to the IL-6 mRNA. In addition, our functional studies confirmed the regulatory role of the interaction between PRINS and IL-6 mRNA. Our results suggest a general anti-inflammatory function for PRINS and provide insight to the role of high PRINS expression in psoriatic non-lesional skin.

## Materials and Methods

### Cell Culture and Inflammatory Stimuli

Normal human epidermal keratinocytes used for the experiments were separated from skin specimens obtained from the Plastic Surgery Unit of our department. Written informed consent was obtained from all investigated individuals. The study was approved by the Human Investigation Review Board of the University of Szeged and complied with the ethical standards of research and in accordance with the Helsinki Declaration.

Epidermis was separated from the dermis by overnight incubation in Dispase (Roche Diagnostics, Manheim, Germany), and keratinocytes were obtained after maceration in 0.25% trypsin. Cells were grown in 75 cm^2^ cell-culture flasks and were maintained in keratinocyte serum-free medium, containing EGF and BPE (Gibco Keratinocyte SFM Kit; Life Technologies, Copenhagen, Denmark) and supplemented with 1% antibiotic/antimycotic solution (PAA Laboratories GmBH, Pasching, Austria) and 1% l-glutamine (PAA Laboratories), at 37°C in a humidified atmosphere with 5% CO_2_. The medium was changed every 2 days.

Third passage keratinocytes were seeded into 6-well plates. Cells were primed in supplement-free medium by addition of 5 ng/ml tumor necrosis factor-α (TNF-α) and 5 ng/ml interferon-γ (IFN-γ). After 24 h, cells were transfected with 0.666 µg/ml polydeoxyadenylic acid–polydeoxythymidylic acid double-stranded homopolymer [poly(dA:dT), Sigma Aldrich, Saint Louis, MO, USA] using the X-tremeGENE 9 DNA transfection reagent (Roche Diagnostics). Cells were harvested 12 h after poly(dA:dT) transfection.

### Gene-Specific Overexpression

For overexpressing PRINS, the AK022045 cDNA sequence [Biological Resource Center (NBRC) National Institute of Technology and Evaluation, Chiba, Japan] was cloned into a pcDNA3.1(+) vector. The empty pcDNA3.1(+) vector served as a control.

The ΔPRINS construct was created by replacing the AK022045 region (position 538–622) with the following scrambled sequence: GTGCGTGGCGGAGACGTGGTGGTAGACCGAATTGAGGAGGATCCGAAGGTTAGACGTAGGCGATCGCCGCTTCGGACGCGGTCGC. The ΔPRINS sequence was created by GeneArt gene synthesis (Thermo Scientific), and cloned into a pcDNA3.1(+) vector.

Transient transfection of NHEK cells was carried out at approximately 70% confluency in parallel to cytokine priming, using the X-tremeGENE HP DNA transfection protocol, as described by the manufacturer (Roche Diagnostics). The transfection efficiency was 85% on average, as determined by the transfection of a GFP reporter construct (Lonza, Basel, Switzerland) and analysis of GFP expression by flow cytometry. The effectiveness of overexpression was investigated with real-time RT-PCR (Figure S1 in Supplementary Material).

### *In Silico* Prediction of Interacting Sites

Sequence complementarity between PRINS (AK022045) and the mRNA of IL-1α (M28983.1), IL-1β (NM_000576.2), IL-6 (NM_000600.4), IL-8 (NM_000584.3), TNF-α (NM_000594.3), and CCL-5 (NM_002985.2) was analyzed using two algorithms: RIsearch (20), which uses a simplified nearest-neighbor energy model, and INTARNA (21–23), which calculates the free-energy values of the interaction based on predicted global and local structures of mRNAs. The regions predicted by both programs were considered as putative interaction sites.

### RNA Isolation and RT-PCR

Total RNA was isolated from cells using TRIzol^®^ Reagent (Invitrogen Corp., Carlsbad, CA, USA), following the manufacturer’s instructions. Turbo DNA-free Kit (Ambion, Life Technologies) was used for the removal of contaminating DNA. cDNA was synthesized from 1 µg total RNA using the iScript cDNA Synthesis Kit (Bio-Rad Laboratories, Hercules, CA, USA). Real-time RT-PCR experiments were carried out with the Universal Probe Library system (Roche Diagnostics) using a C1000 Touch Thermal Cycler (Bio-Rad Laboratories) and the primers listed in Table S1 in Supplementary Material. The expression of each gene was normalized to the 18S rRNA gene. Relative mRNA levels were calculated by the ΔΔCt method.

### ELISA

Cell supernatants were centrifuged (5,000 rpm, 4 min, 4°C) to pellet cell debris, and the amount of cytokines IL-1α, IL-1β, IL-6, IL-8, CCL-5, and TNF-α was determined by ELISA (IL-1α Duo Set, IL-1β ELISA Duo Set, and TNF-α Duo Set, R&D Systems, Minneapolis, MN, USA; Human IL-6 Mini TMB ELISA Development Kit, Human IL-8 TMB ELISA Development Kit, Human RANTES ATBS ELISA Development Kit, PeproTech, Rocky Hill, NJ, USA), according to the manufacturer’s instructions.

### *In Vitro* Transcription and *In Vitro* Binding Assay

PRINS and ΔPRINS RNA sequences were produced by *in vitro* transcription from pcDNA3.1(+) containing the AK022045 or ΔPRINS cDNA sequence, using Transcript Aid T7 *In Vitro* Transcription Kit (Thermo Scientific). Products were purified by the GeneJET RNA Purification Kit (Thermo Scientific). Quality and sequence-length analyses were carried out on reducing agarose gel-electrophoresis. The single-stranded RNA products were used in a fluorescent binding assay. The fluorescently labeled RNA sequence 5′6-carboxyfluorescein(6-FAM)/GAAGCUCUAUCUCCCCUCCAGGAGCCCAGCUAUGAACUCCUUCUCCACAAGCGCCUUCGGUCCAGUUGCCUUCUCCCUGGGGCUGCUCCUGGUGUUGCCUGCUGCCUUCCCUGCC-3′, comprising positions 91–205 of the IL-6 (NM_000600.4) mRNA sequence, was produced by Integrated DNA Technologies.

An *in vitro* binding assay was carried out on a Monolith NT.115 Pico MicroScale Thermophoresis instrument (NanoTemper GmbH, Germany), in nuclease-free water, at 25°C, with 80% Laser Power, 10% LED Power, by 2bind GmbH, Regensburg, Germany. Fluorescence enhancement of the 6-FAM labeled specific truncated IL-6 RNA sequence or a 6-FAM labeled unspecific DNA, as negative control, was measured after addition of PRINS or ΔPRINS. The concentration of fluorescently labeled molecules was 10 nM constantly, while unlabeled RNA concentration ranged from 126.75 nM to 61.9 pM. Initial fluorescence was analyzed for binding curves; the formulation used is displayed in Figure S2 in Supplementary Material.

### Statistical Analysis

Experiments were carried out in triplicate with at least three biological repeats, as indicated in figure legends. For statistical analysis, one-way ANOVA was used to compare more than two groups, and one-tailed, paired *t*-test was used to compare two groups. Statistical analysis was carried out using R software, version 3.2.2., and the significance level was set at *p* ≤ 0.05.

## Results

### Poly(dA:dT) Treatment of Keratinocytes Induces the Expression of Inflammatory Cytokines while Decreasing the Expression of PRINS

**Figure 1:**
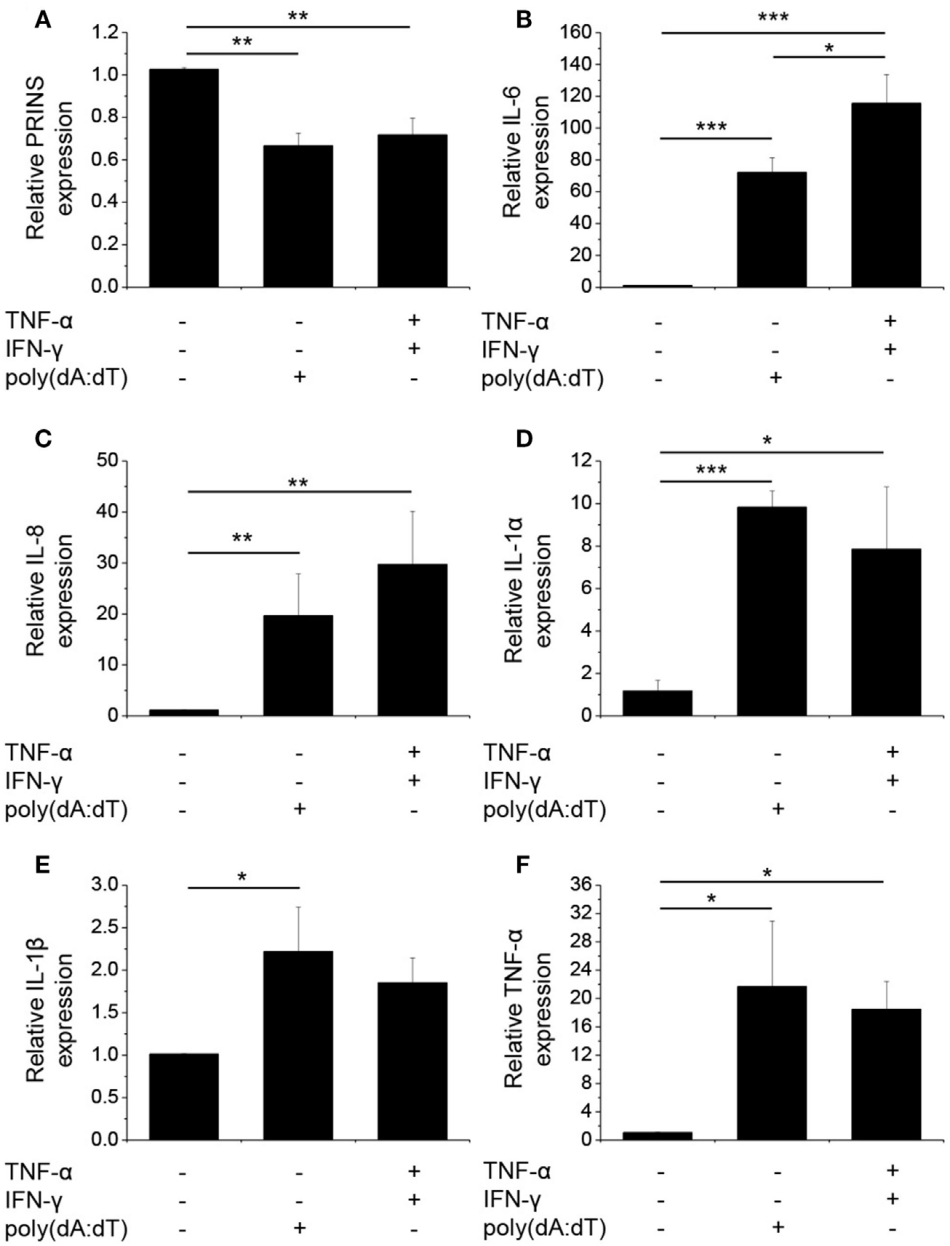
Poly(dA:dT) treatment decreased the expression of PRINS but induced the expression and secretion of cytokines. Normal human epidermal keratinocytes were transfected with poly(dA:dT) for 12 h with or without priming for 24 h with 5 ng/ml tumor necrosis factor-α (TNF)-α and interferon-γ (IFN-γ). RNA expression was detected for PRINS **(A)** and cytokines interleukin-6 (IL-6) **(B)**, IL-8 **(C)**, IL-1α **(D)**, IL-1β **(E)**, TNF-α **(F)** by real-time RT-PCR. Data are presented as mean ± SE of five independent experiments (*p ≤ 0.05; **p ≤ 0.01; ***p ≤ 0.001).

**Figure 2:**
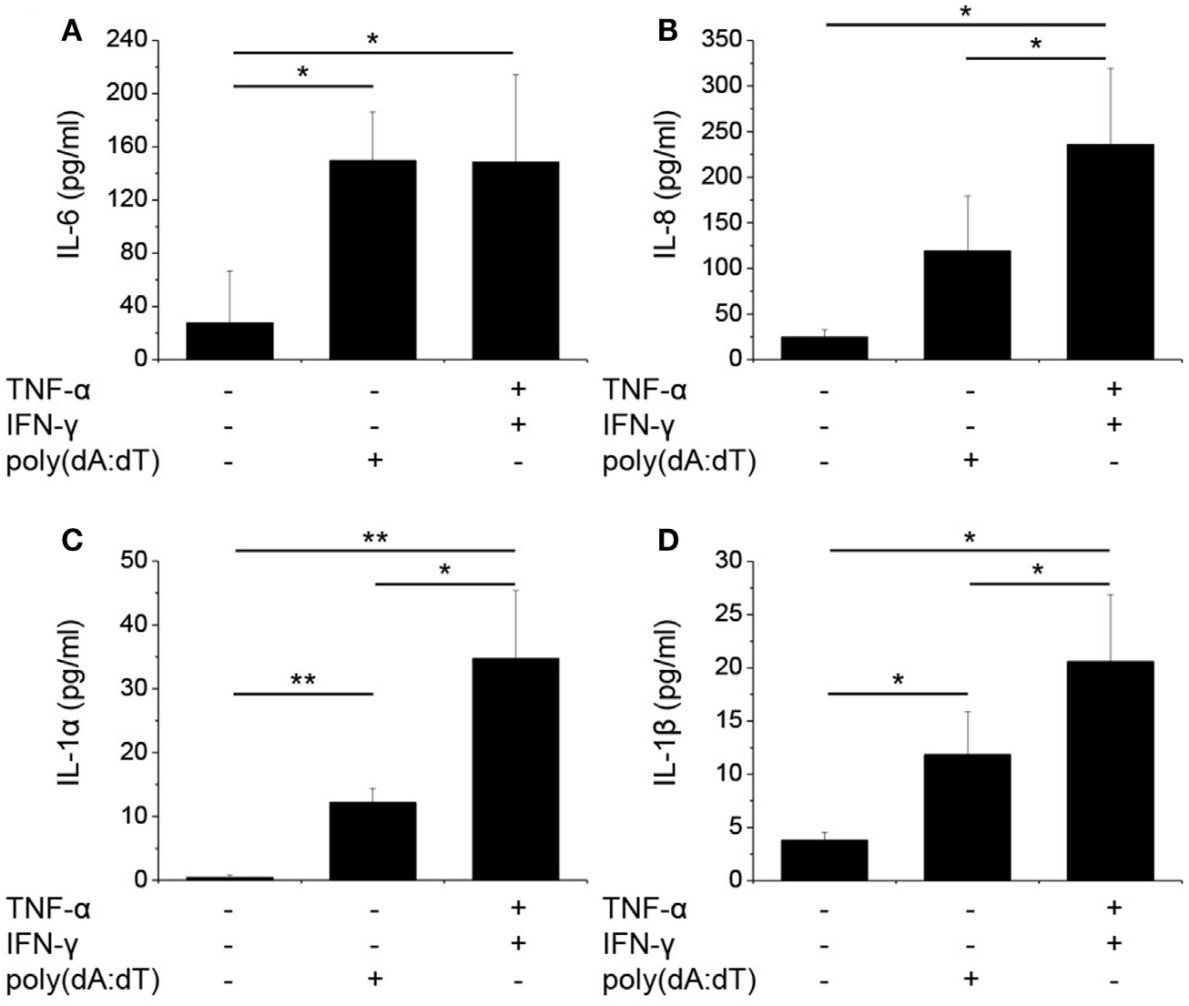
Poly(dA:dT) treatment induced the secretion of cytokines by normal human epidermal keratinocytes (NHEKs). NHEKs were transfected with poly(dA:dT) for 12 h with or without priming for 24 h with 5 ng/ml tumor necrosis factor-α (TNF-α) and interferon-γ (IFN-γ). Secretion of cytokines interleukin-6 (IL-6) **(A)**, IL-8 **(B)**, IL-1α **(C)**, IL-1β **(D)** was detected by ELISA from cell supernatant. Data are presented as mean ± SE of five independent experiments (*p ≤ 0.05; **p ≤ 0.01; ***p ≤ 0.001).

To study how dsDNA influences NHEKs immune function, we analyzed the expression (Figure 1) and secretion (Figure 2) of several inflammatory cytokines and the expression of PRINS upon poly(dA:dT) exposure.

We confirmed that poly(dA:dT) significantly induced the expression and secretion of IL-1β in keratinocytes, as described previously (4). In addition, poly(dA:dT) induced very strong mRNA expression of IL-1α, IL-6, IL-8, and TNF-α in NHEKs (Figures 1B–F).

To gain pronounced inflammatory reaction in NHEKs, a priming step using TNF-α and IFN-γ is often applied before poly(dA:dT) treatment (3, 4); however, the cumulative effect of this treatment on the expression of other cytokines has not been examined thoroughly. When cells were primed for 24 h before poly(dA:dT) treatment, IL-1β secretion was enhanced by the combination of TNF-α and IFN-γ compared to poly(dA:dT) treatment alone. In contrast, IL-1β mRNA expression was not affected by priming (Figures 1E and 2D). Similarly, priming had no effect on TNF-α mRNA expression (Figure 1F). However, priming induced significantly higher mRNA expression of the other investigated cytokine genes (Figures 1B–D).

In a manner similar to mRNA expression, poly(dA:dT) transfection induced the secretion of the investigated cytokines, and the priming step significantly enhanced the amount of secreted IL-1α, IL-1β, and IL-8 in the keratinocyte supernatant (Figure 2).

As we and others have demonstrated altered PRINS expression upon exposure to inflammatory molecules (14, 16), we examined PRINS expression upon poly(dA:dT) treatment. Poly(dA:dT) alone or combined with cytokine priming decreased the expression of PRINS in NHEKs (Figure 1A). The expression of PRINS returned to the initial level after 24 h (data not shown), whereas the expression of inflammatory cytokines declined during this period.

### PRINS Overexpression Decreased IL-6 and IL-8 Levels in Keratinocytes

**Figure 3:**
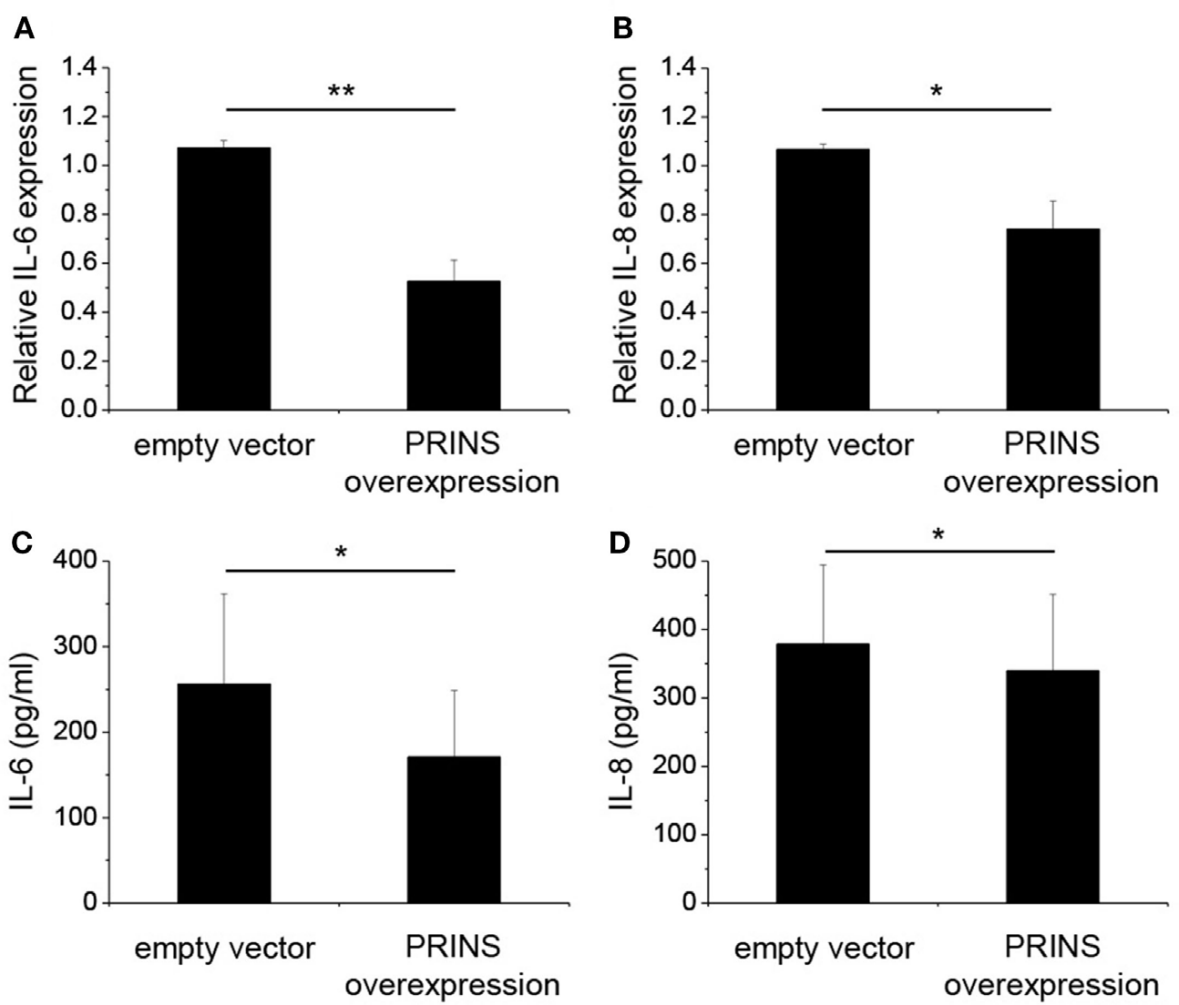
Overexpression of PRINS regulates the expression and secretion of interleukin-6 (IL-6) and IL-8. In parallel to priming with 5 ng/ml tumor necrosis factor-α and interferon-γ, normal human epidermal keratinocytes were transfected with a pcDNA3.1(+) vector containing the PRINS cDNA; an empty pcDNA3.1(+) vector was used as control. After 24 h, cells were transfected with poly(dA:dT). RNA expression of cytokines IL-6 **(A)** and IL-8 **(B)** was detected by real-time RT-PCR. Secretion of cytokines IL-6 **(C)** and IL-8 **(D)**, was detected by ELISA from cell supernatants. Data are presented as mean ± SE of four independent experiments (*p ≤ 0.05; **p ≤ 0.01).

The change in PRINS expression upon inflammatory stimuli suggests the possible contribution of PRINS to inflammatory responses of NHEK cells. To determine whether PRINS can regulate inflammatory cytokine expression, cells were transfected with a construct for overexpressing PRINS during the combined priming and poly(dA:dT) treatment. Expression and secretion of IL-6 and IL-8 was significantly decreased by PRINS overexpression (Figure 3), whereas mRNA expression and secretion of IL-1α, IL-1β, and TNF-α were not affected (data not shown). These results suggest that PRINS does not influence inflammasome activation, but instead influences the regulation of other inflammatory processes.

### *In Silico* Analysis Revealed Putative Interacting Sites between the PRINS lncRNA and the IL-6 mRNA

The mRNA of the chemokine CCL-5 was previously predicted to interact with PRINS; however, it was not reported whether this interaction affects the stability of the CCL-5 mRNA (15). Therefore, we measured mRNA expression and secretion of CCL-5 during PRINS overexpression, and found that both decreased (Figure S3 in Supplementary Material) in a manner similar to the changes observed for IL-6 and IL-8. The similarity of these expression profiles led us to hypothesize similar mechanism(s) for IL-6, IL-8, and CCL-5 regulation mediated by PRINS.

**Figure 4:**
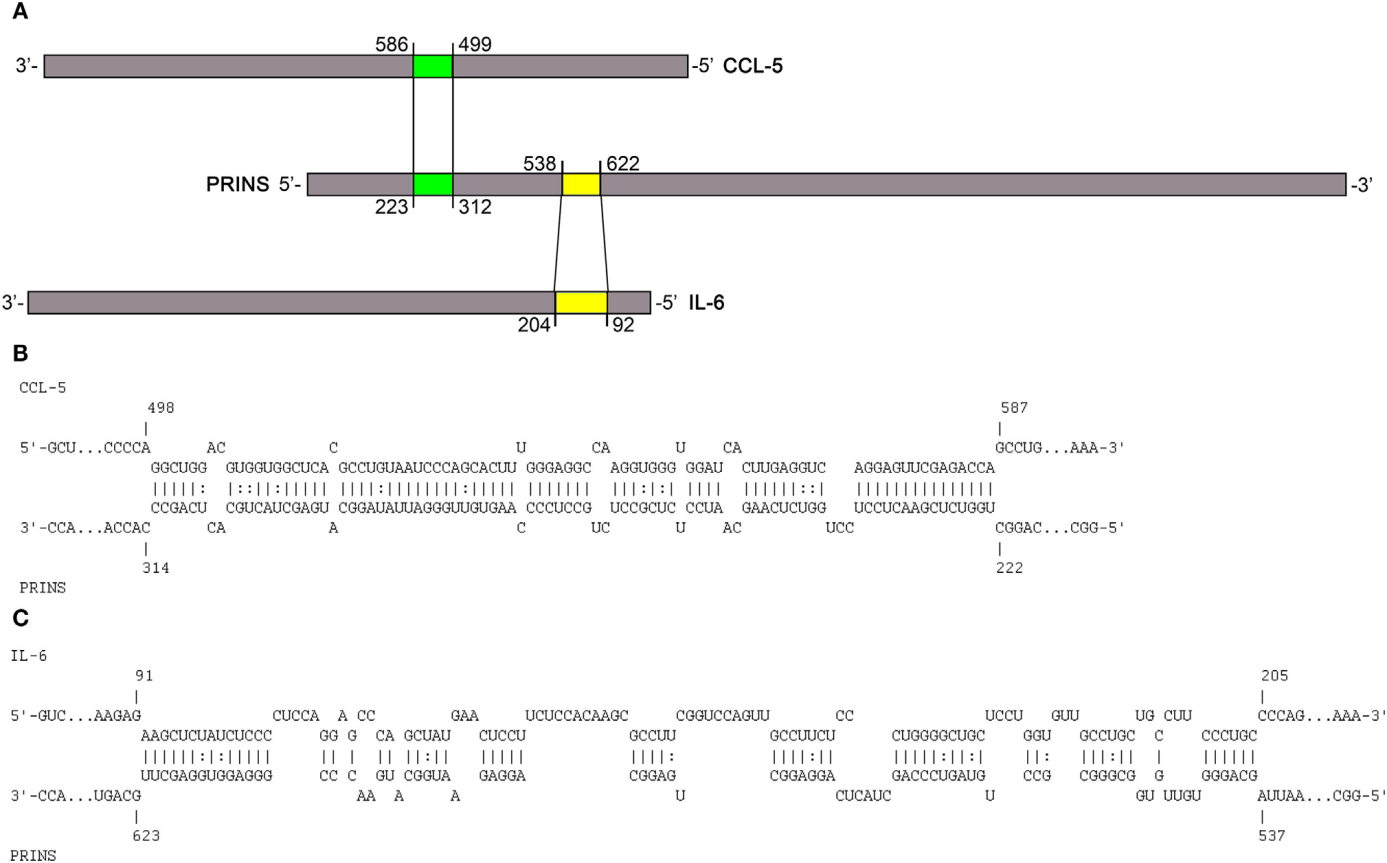
Putative interactions between PRINS long non-coding RNA and chemokine (C-C motif) ligand 5 (CCL-5) and interleukin-6 (IL-6) mRNAs. Schematic representation of the *in silico* predicted interaction sites in the PRINS sequence for the CCL-5 and IL-6 mRNAs **(A)**. PRINS sequences predicted to interact with CCL-5 mRNA **(B)** and IL-6 mRNA **(C)**. Nucleotide positions are given based on the AK022045, NM_000600.4, and NM_002985.2 reference sequences.

To predict interactions between PRINS (AK022045) and the mRNAs of IL-6 (NM_00600.4) and IL-8 (NM_000584.3), we performed an *in silico* analysis using INTARNA (22, 23) and RIsearch (20) software. As sequence details of the CCL-5 mRNA and PRINS interaction have not been described in detail (15), we also included the CCL-5 mRNA (NM_002985.2) in the *in silico* analysis. As a control for the reliability of the prediction analyses, mRNA sequences of cytokines not affected by PRINS overexpression (IL-1α—M28983.1, IL-1β—NM_000576.2, TNF-α—NM_000594.3) were also included. The regions predicted by both algorithms were considered putative interacting sites. Putative interaction sites were not predicted for the cytokines not affected by PRINS overexpression (IL-1α, IL-1β, TNF-α) in this analysis. PRINS interaction regions were only predicted for the IL-6 and CCL-5 mRNAs (Figures 4B,C), two mediators affected by PRINS overexpression. A distance of approximately 200 nt separates the predicted interaction sites in the PRINS sequence, and the corresponding sites occur in the 5′ untranslated region (UTR) of IL-6 and the 3′ UTR of CCL-5 (Figure 4A). No interaction site was predicted for IL-8.

### PRINS Binds to IL-6 mRNA through Direct, Sequence-Specific Interaction

**Figure 5:**
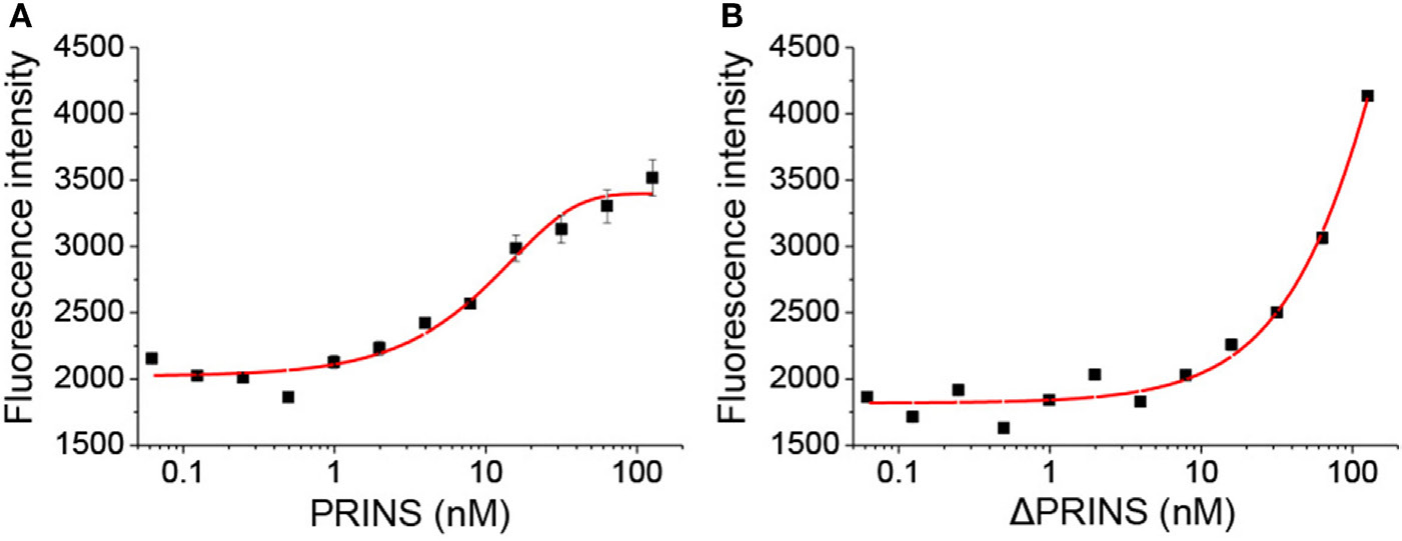
PRINS specifically binds to interleukin-6 (IL-6) mRNA. The binding affinity of PRINS **(A)** and ΔPRINS **(B)** to IL-6 mRNA was determined by analyzing the initial fluorescence enhancement caused by specific binding. The concentration of labeled IL-6 mRNA was constant (10 nM), while the concentration of PRINS and ΔPRINS varied from 62 pM to 127 nM.

To validate the predicted interaction site, an *in vitro* binding experiment was carried out using the PRINS lncRNA and the IL-6 mRNA. Binding affinity was determined using the single-stranded PRINS RNA and a fluorescently labeled, truncated IL-6 mRNA sequence containing the predicted interacting sequence. The ΔPRINS sequence, in which the predicted interaction site to IL-6 was replaced by scrambled sequence, was used as a control. As a negative control, a fluorescently labeled DNA sequence with no similarity to either PRINS or IL-6 RNAs was used. While PRINS exhibited a very high binding affinity to the labeled IL-6 mRNA (Figure 5A, Kd = 10.3436 nM), specific binding for ΔPRINS and the unspecific labeled DNA could not be detected (Figure 5B). This result confirms *in vitro* the specificity of the regions predicted *in silico* (538–622 of PRINS, AK022045 and 92–204 of IL-6 mRNA, NM_000600.4).

### PRINS Decreases IL-6 Expression in NHEKs through Sequence-Specific Interaction

**Figure 6:**
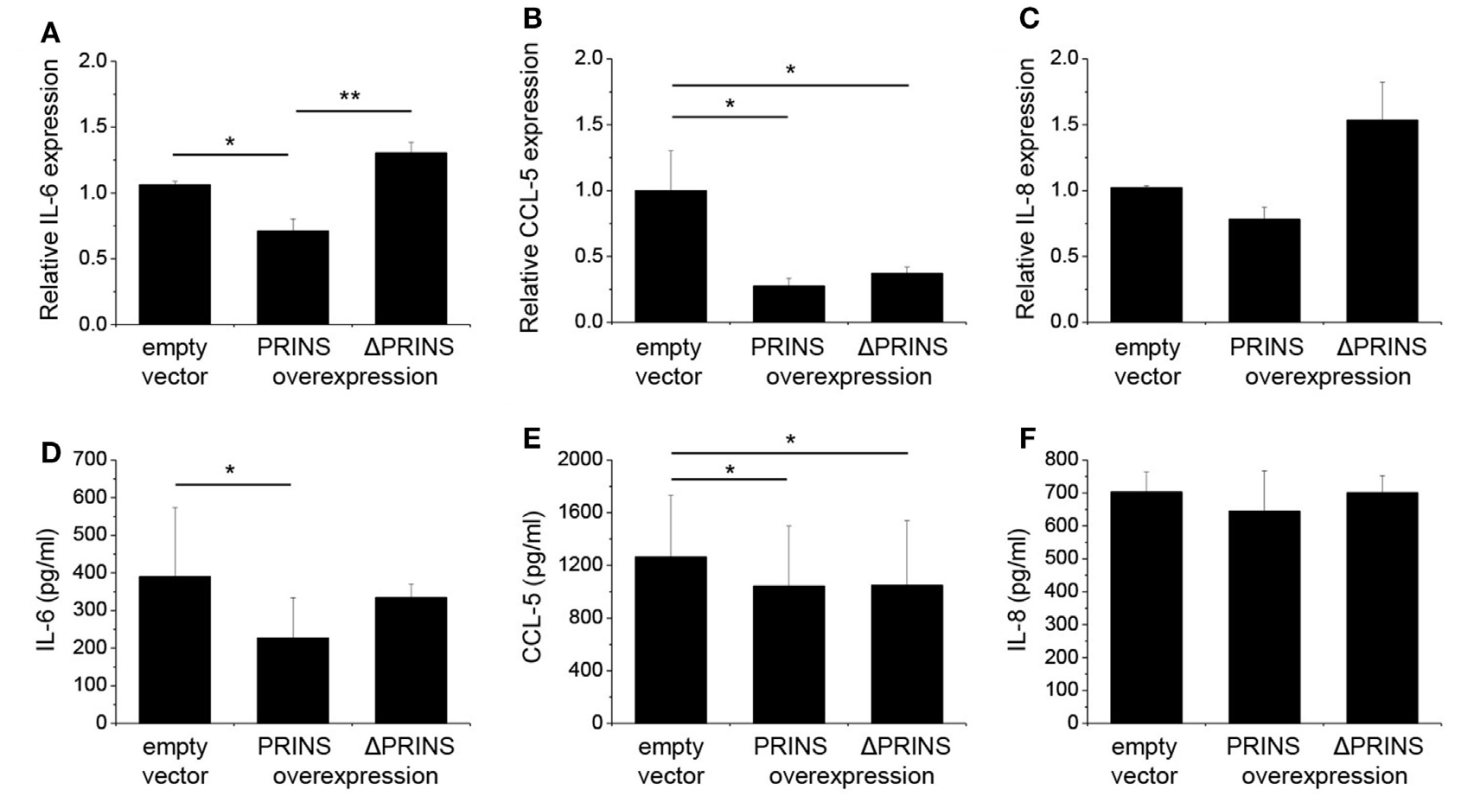
The 538–622 nt region of PRINS is required for the regulation of interleukin-6 (IL-6) expression. In parallel to priming with 5 ng/ml tumor necrosis factor-α and interferon-γ, normal human epidermal keratinocytes were transfected with a pcDNA3.1(+) vector containing the PRINS cDNA or the ΔPRINS cDNA (containing a scrambled version of the IL-6 binding site at positions 538–622 nt); the empty pcDNA3.1(+) vector served as control, after 24 hours cells were transfected by poly(dA:dT). Expression of cytokines IL-6 **(A)**, CCL-5 **(B)**, and IL-8 **(C)** was detected by real-time RT-PCR. Secretion of cytokines IL-6 **(D)**, CCL-5 **(E)**, and IL-8 **(F)** was detected by ELISA from cell supernatants. Nucleotide positions based on the AK022045 reference sequence. Data are presented as mean ± SE of three independent experiments (*p ≤ 0.05; **p ≤ 0.01).

To further validate the functionality of the *in silico* predicted and *in vitro* determined IL-6 mRNA interacting region in the PRINS sequence, we performed the overexpression experiments in NHEKs with vectors containing the wild-type PRINS or ΔPRINS (with scrambled IL-6 binding site) sequences, during the combined priming and poly(dA:dT) treatment. IL-6 expression was not affected by overexpression of ΔPRINS but was, in contrast, significantly decreased by PRINS overexpression (Figure 6A), and similar tendencies were seen in the amount of secreted IL-6 (Figure 6D). To confirm the specificity of this region in IL-6 regulation, the expression of CCL-5 was also studied. CCL-5 expression and secretion decreased similarly both in cells overexpressing ΔPRINS and in cells overexpressing PRINS (Figures 6B,E). IL-8 expression upon PRINS or ΔPRINS overexpression showed a similar tendency to IL-6 expression, although significant differences could not be detected (Figures 6C,F). These result demonstrated that the binding site in the PRINS sequence is essential and specific for the regulation of IL-6 expression.

## Discussion

Due to large-scale gene-expression studies the number of annotated human non-coding RNAs has increased rapidly (24), but functional roles have been assigned only to a few of them (25, 26). PRINS was one of the first lncRNAs described to be dysregulated in a disease, namely in psoriasis (13). PRINS is located in an intron of the KIAA1217 gene on the sense strand (27), and, thus, it can be considered as a long intronic non-coding RNA. The expression of PRINS increases in response to various cellular stressors (14) in HaCaT cells, as well as to hypoxia in HK-2 cells (15) suggesting that PRINS contributes to the stress response of the cells. Recent reports on PRINS showing decreased expression upon inflammatory stimuli in both NHEKs and macrophages (14, 16), and the potential interaction between PRINS and the mRNA of CCL-5 (15) has led us to study its potential contribution to keratinocytes immune responses.

To study keratinocyte immune functions, we applied a treatment with the cytosolic DNA-analog poly(dA:dT), which was reported to induce inflammasome activation in these cells (3, 28) and induce the expression of several cytokines through a RIG-I-dependent mechanism in professional immune cells (29). Before poly(dA:dT) treatment, a priming with the cytokines TNF-α and IFN-γ was applied, which were described to be key elements in keratinocyte immune responses (30) and induce a pronounced inflammasome activation (4). Our results are in agreement with recent reports describing inflammasome activation in keratinocytes (3, 4, 28) and IL-6, IL-8, and TNF-α induction in canine keratinocytes (6) and human melanocytes (31) upon poly(dA:dT) exposition. Priming induced significantly enhanced mRNA expression of IL-6 and IL-8. In addition, cytokine secretion significantly increased compared to poly(dA:dT) treatment alone. In contrast to cytokine expression, poly(dA:dT) treatment decreased PRINS expression in NHEKs in line with previous reports in NHEKs and macrophages (14, 16).

PRINS was recently described to potentially interact with the CCL-5 mRNA (15), but its function remains to be elucidated. In this study, we demonstrate that PRINS overexpression decreases the expression of IL-6, IL-8, and CCL-5 in keratinocytes upon poly(dA:dT) treatment. In addition, decreased IL-6 expression was observed upon PRINS overexpression in UVB-treated samples as well (data not shown). Using *in silico* analysis, regions in the PRINS sequence were predicted to interact with CCL-5 and IL-6 mRNA. As inhibition of transcription by DNA:lncRNA triplex formation or posttranscriptional destabilization of the mRNA by mRNA–lncRNA duplex formation has been reported (25), we analyzed the sequences and found that the interacting site on IL-6 spans two exons, indicating an mRNA–lncRNA interaction. Moreover, PRINS demonstrated perinuclear localization in keratinocytes (32), making it possible to exert its effect at the posttranscriptional level. The mRNA–lncRNA interaction was validated *in vitro*: PRINS showed a very high binding affinity (Kd = 10.3436 nM) to IL-6 mRNA, and the destruction of the predicted binding site abolished the ability of PRINS to bind to the IL-6 mRNA. The binding site of PRINS lies within the 5′ UTR of the IL-6 mRNA, which is a rare phenomenon. The majority (~40%) of lncRNAs bind to the 3′ UTR of their target mRNAs, while only around 5% of lncRNAs is able to bind to the 5′ UTR of their target (33). The functionality of the mRNA–lncRNA interaction was also observed at the cellular level, as overexpression of ΔPRINS, in which the IL-6 interacting site is scrambled, did not decrease IL-6 levels. Thus, we demonstrated that PRINS is able to bind the IL-6 mRNA and this specific interaction destabilizes IL-6 expression and secretion in NHEKs.

Similar to IL-6, PRINS overexpression decreased the expression of IL-8, but no interaction site could be predicted. We hypothesize that this is a secondary effect (34); however, we cannot exclude the possibility of a yet unknown interaction site between PRINS and IL-8 mRNA.

Many lncRNAs which were reported to regulate mRNA expression were found to act as primary transcripts for miRNAs (35). We have found no evidence that PRINS is cleaved to smaller miRNA precursors, but the IL-6 binding site of PRINS is 96% similar to the miR5585, which is predicted by TargetMiner to have IL-6 mRNA as a target (36). miR5585 is located on Chromosome 7, whereas PRINS is located on Chromosome 10, so their high similarity and overlapping functions might originate from gene duplication.

Like many other non-coding RNAs, PRINS seems to have multiple cellular functions (37). It binds to nucleophosmin, a chaperon protein, and facilitates its transition from the nucleolus to the nucleoplasm upon UVB irradiation (32). Additionally, our recent results suggest that PRINS is involved with inflammation by inhibiting cytokine expression. Based on our current and previous findings (13–15), we hypothesize that elevated PRINS expression in psoriatic uninvolved epidermis may contribute to downregulation of the inflammatory functions in psoriatic keratinocytes and maintenance of normal phenotype.

Our studies were performed using a non-professional immune cell type, keratinocytes; however, the same mechanisms might be also relevant in professional immune cells. Further studies on the same PRINS-related cellular events upon additional stressors such as UVB or microbial components may widen our knowledge on the cellular functions of PRINS and, in general, about lncRNAs.

## Author Contributions

JD and MS designed the study, JD and AG prepared laboratory work and analyzed the data. DJ, AG, ZB-C, LK, and MS interpreted the data and drafted the manuscript. All authors approved the final version.

## Conflict of Interest Statement

The authors declare that the research was conducted in the absence of any commercial or financial relationships that could be construed as a potential conflict of interest.

## References

[1] Luff JA Yuan H Kennedy D Schlegel R Felsburg P Moore PF. Keratinocyte antiviral response to poly(dA:dT) stimulation and papillomavirus infection in a canine model of X-linked severe combined immunodeficiency. PLoS One (2014) 9:e102033. 10.1371/journal.pone.0102033 25025687 PMC4099134

[2] Suspène R Mussil B Laude H Caval V Berry N Bouzidi MS Self-cytoplasmic DNA upregulates the mutator enzyme APOBEC3A leading to chromosomal DNA damage. Nucleic Acids Res (2017) 45:3231–41. 10.1093/nar/gkx001 28100701 PMC5389686

[3] Dombrowski Y Peric M Koglin S Kammerbauer C Göss C Anz D Cytosolic DNA triggers inflammasome activation in keratinocytes in psoriatic lesions. Sci Transl Med (2011) 3:82ra38. 10.1126/scitranslmed.3002001 21562230 PMC3235683

[4] Göblös A Danis J Vas K Bata-Csörgő Z Kemény L Széll M. Keratinocytes express functional CARD18, a negative regulator of inflammasome activation, and its altered expression in psoriasis may contribute to disease pathogenesis. Mol Immunol (2016) 73:10–8. 10.1016/j.molimm.2016.03.009 27023378

[5] Prens EP Kant M van Dijk G van der Wel LI Mourits S van der Fits L. IFN-alpha enhances poly-IC responses in human keratinocytes by inducing expression of cytosolic innate RNA receptors: relevance for psoriasis. J Invest Dermatol (2008) 128:932–8. 10.1038/sj.jid.5701087 17928888

[6] Luff JA Yuan H Suter MM Müller EJ Schlegel R Moore PF. Canine keratinocytes upregulate type I interferons and proinflammatory cytokines in response to poly(dA: dT) but not to canine papillomavirus. Vet Immunol Immunopathol (2013) 153:177–86. 10.1016/j.vetimm.2013.02.001 23557936 PMC4425420

[7] Lowes MA Suárez-Fariñas M Krueger JG. Immunology of psoriasis. Annu Rev Immunol (2014) 32:227–55. 10.1146/annurev-immunol-032713-120225 24655295 PMC4229247

[8] Lande R Gregorio J Facchinetti V Chatterjee B Wang Y-H Homey B Plasmacytoid dendritic cells sense self-DNA coupled with antimicrobial peptide. Nature (2007) 449:564–9. 10.1038/nature06116 17873860

[9] Tsoi LC Iyer MK Stuart PE Swindell WR Gudjonsson JE Tejasvi T Analysis of long non-coding RNAs highlights tissue-specific expression patterns and epigenetic profiles in normal and psoriatic skin. Genome Biol (2015) 16:24. 10.1186/s13059-014-0570-4 25723451 PMC4311508

[10] Li B Tsoi LC Swindell WR Gudjonsson JE Tejasvi T Johnston A Transcriptome analysis of psoriasis in a large case-control sample: RNA-seq provides insights into disease mechanisms. J Invest Dermatol (2014) 134:1828–38. 10.1038/jid.2014.28 24441097 PMC4057954

[11] Sonkoly E Wei T Janson PCJ Sääf A Lundeberg L Tengvall-Linder M MicroRNAs: novel regulators involved in the pathogenesis of psoriasis? PLoS One (2007) 2:1–8. 10.1371/journal.pone.0000610 PMC1905940 17622355

[12] Maass PG Luft FC Bähring S. Long non-coding RNA in health and disease. J Mol Med (2014) 92:337–46. 10.1007/s00109-014-1131-8 24531795

[13] Sonkoly E Bata-Csörgő Z Pivarcsi A Polyánka H Kenderessy-Szabó A Molnár G Identification and characterization of a novel, psoriasis susceptibility-related noncoding RNA gene, PRINS. J Biol Chem (2005) 280:24159–67. 10.1074/jbc.M501704200 15855153

[14] Bari L Bacsa S Sonkoly E Bata-Csörgő Z Kemény L Dobozy A Comparison of stress-induced PRINS gene expression in normal human keratinocytes and HaCaT cells. Arch Dermatol Res (2011) 303:745–52. 10.1007/s00403-011-1162-8 21750967

[15] Yu T-M Palanisamy K Sun K-T Day Y-J Shu K-H Wang I-K RANTES mediates kidney ischemia reperfusion injury through a possible role of HIF-1α and LncRNA PRINS. Sci Rep (2016) 6:18424. 10.1038/srep18424 26725683 PMC4698731

[16] Pawar K Hanisch C Palma Vera SE Einspanier R Sharbati S. Down regulated lncRNA MEG3 eliminates mycobacteria in macrophages via autophagy. Sci Rep (2016) 6:19416. 10.1038/srep19416 26757825 PMC4725832

[17] Ouwehand K Spiekstra SW Waaijman T Breetveld M Scheper RJ de Gruijl TD CCL5 and CCL20 mediate immigration of Langerhans cells into the epidermis of full thickness human skin equivalents. Eur J Cell Biol (2012) 91:765–73. 10.1016/j.ejcb.2012.06.004 22857950

[18] de Groot M Teunissen MBM Ortonne JP Lambert JR Naeyaert JM Picavet DI Expression of the chemokine receptor CCR5 in psoriasis and results of a randomized placebo controlled trial with a CCR5 inhibitor. Arch Dermatol Res (2007) 299:305–13. 10.1007/s00403-007-0764-7 17647003 PMC1950346

[19] Giustizieri ML Mascia F Frezzolini A De Pità O Chinni LM Giannetti A Keratinocytes from patients with atopic dermatitis and psoriasis show a distinct chemokine production profile in response to T cell-derived cytokines. J Allergy Clin Immunol (2001) 107:871–7. 10.1067/mai.2001.114707 11344355

[20] Wenzel A Akbasli E Gorodkin J. RIsearch: fast RNA-RNA interaction search using a simplified nearest-neighbor energy model. Bioinformatics (2012) 28:2738–46. 10.1093/bioinformatics/bts519 22923300 PMC3476332

[21] Smith C Heyne S Richter AS Will S Backofen R. Freiburg RNA tools: a web server integrating INTARNA, EXPARNA and LOCARNA. Nucleic Acids Res (2010) 38:W373–7. 10.1093/nar/gkq316 20444875 PMC2896085

[22] Busch A Richter AS Backofen R. IntaRNA: efficient prediction of bacterial sRNA targets incorporating target site accessibility and seed regions. Bioinformatics (2008) 24:2849–56. 10.1093/bioinformatics/btn544 18940824 PMC2639303

[23] Wright PR Georg J Mann M Sorescu DA Richter AS Lott S CopraRNA and IntaRNA: predicting small RNA targets, networks and interaction domains. Nucleic Acids Res (2014) 42:W119–23. 10.1093/nar/gku359 24838564 PMC4086077

[24] Derrien T Johnson R Bussotti G Tanzer A Djebali S Tilgner H The GENCODE v7 catalog of human long noncoding RNAs: analysis of their gene structure, evolution, and expression. Genome Res (2012) 22:1775–89. 10.1101/gr.132159.111 22955988 PMC3431493

[25] Kung JTY Colognori D Lee JT. Long noncoding RNAs: past, present, and future. Genetics (2013) 193:651–69. 10.1534/genetics.112.146704 23463798 PMC3583990

[26] Heward JA Lindsay MA Long non-coding RNAs in the regulation of the immune response. Trends Immunol (2014) 35:408–19. 10.1016/j.it.2014.07.005 25113636 PMC7106471

[27] Széll M Danis J Bata-Csörgő Z Kemény L. PRINS, a primate-specific long non-coding RNA, plays a role in the keratinocyte stress response and psoriasis pathogenesis. Pflügers (2016) 468:935–43. 10.1007/s00424-016-1803-z 26935426 PMC4893059

[28] Kopfnagel V Wittmann M Werfel T. Human keratinocytes express AIM2 and respond to dsDNA with IL-1β secretion. Exp Dermatol (2011) 20:1027–9. 10.1111/j.1600-0625.2011.01382.x 22092578

[29] Ablasser A Bauernfeind F Hartmann G Latz E Fitzgerald KA Hornung V RIG-I dependent sensing of poly(dA-dT) via the induction of an RNA polymerase III transcribed RNA intermediate. Nat Immunol (2009) 10:1065–72. 10.1038/ni.1779 19609254 PMC3878616

[30] Szabó K Bata-Csörgő Z Dallos A Bebes A Francziszti L Dobozy A Regulatory networks contributing to psoriasis susceptibility. Acta Derm Venereol (2014) 94:380–5. 10.2340/00015555-1708 24419088

[31] Wang S Liu D Ning W Xu A. Cytosolic dsDNA triggers apoptosis and pro-inflammatory cytokine production in normal human melanocytes. Exp Dermatol (2015) 24:298–300. 10.1111/exd.12621 25515776

[32] Szegedi K Göblös A Bacsa S Antal M Németh IB Bata-Csörgő Z Expression and functional studies on the noncoding RNA, PRINS. Int J Mol Sci (2013) 14:205–25. 10.3390/ijms14010205 PMC3565259 23344029

[33] Szcześniak MW Makałowska I. lncRNA-RNA interactions across the human transcriptome. PLoS One (2016) 11:e0150353. 10.1371/journal.pone.0150353 26930590 PMC4773119

[34] Scheller J Chalaris A Schmidt-Arras D Rose-John S. The pro- and anti-inflammatory properties of the cytokine interleukin-6. Biochim Biophys Acta (2011) 1813:878–88. 10.1016/j.bbamcr.2011.01.034 21296109

[35] Gutschner T Diederichs S The hallmarks of cancer: a long non-coding RNA point of view. RNA Biol (2012) 9:703–19. 10.4161/rna.20481 22664915 PMC3495743

[36] Bandyopadhyay S Mitra R. TargetMiner: microRNA target prediction with systematic identification of tissue-specific negative examples. Bioinformatics (2009) 25:2625–31. 10.1093/bioinformatics/btp503 19692556

[37] Kapusta A Feschotte C. Volatile evolution of long noncoding RNA repertoires: mechanisms and biological implications. Trends Genet (2014) 30:439–52. 10.1016/j.tig.2014.08.004 25218058 PMC4464757

