# Supplementary material for: PRINS Non-Coding RNA Regulates Nucleic Acid-Induced Innate Immune Responses of Human Keratinocytes
Source: Front Immunol. 2017 Aug 29;8:1053. doi: 10.3389/fimmu.2017.01053 (PMC5581800; doi:10.3389/fimmu.2017.01053)

Supplementary Table 1. List of primers and probes used for real-time RT-PCR.

				Probe no.
Roche Diagnostics - Universal Probe Library	18S rRNS	Forward	CGC TCC ACC AAC TAA GAA CG	77
		Reverse	CTC AAC ACG GGA AAC CTC AC	
	TNF-α	Forward	CAG CCT CTT CTC CTT CCT GAT	29
		Reverse	GCC AGA GGG CTG ATT AGA GA	
	IL-1α	Forward	GGT TGA GTT TAA GCC AAT CCA	6
		Reverse	TGC TGA CCT AGG CTT GAT GA	
	IL-1β	Forward	AAA GCT TGG TGA TGT CTG GTC	10
		Reverse	AAA GGA CAT GGA GAA CAC CAC T	
	IL-6	Forward	CAG GAG CCC AGC TAT GAA CT	45
		Reverse	GAA GGC AGC AGG CAA CAC	
	CCL5	Forward	TGCCCACATCAAGGAGTATTT	59
		Reverse	CTTTCGGGTGACAAAGACG	

Thermo Fischer Scientific	IL-8	TaqMan Gene Expression Assays	Hs00174103_m1
	PRINS	TaqMan Gene Expression Assays	Hs03671803_s1

Supplementary figure 1. The evaulation of the effectiveness of PRINS overexpression by real-time RT-PCR. NHEKs were transfected with a pcDNA3.1(+) vector containing the PRINS cDNA, while the empty pcDNA3.1(+) vector was used as control.

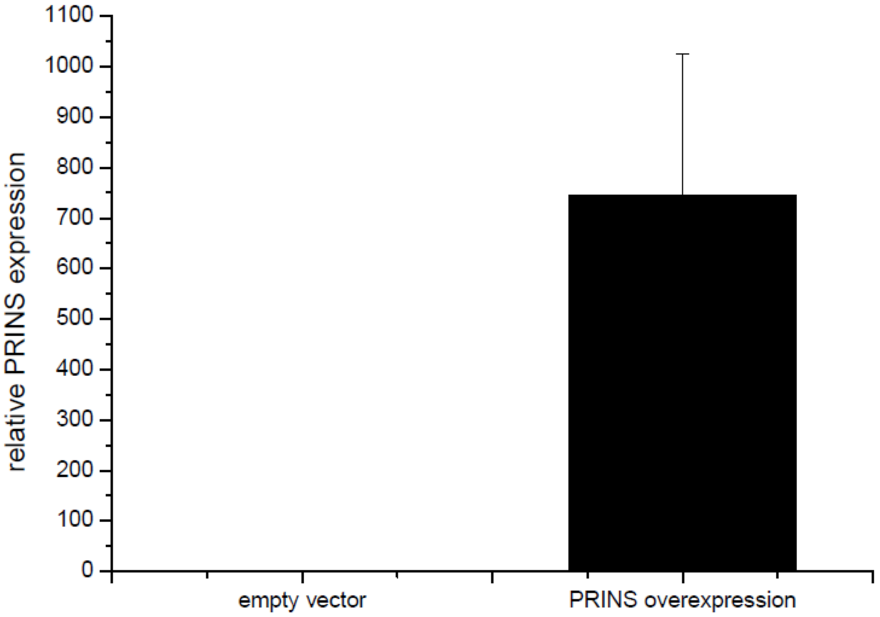

Supplementary figure 2. Curve fit formula used to calculate binding affinities of PRINS and IL-6 based on initial fluorescence.

1:1 binding model:

$$A + T \Leftrightarrow AT$$

$$F(c_T) = F_u + (F_b - F_u) * \frac{c_{AT}}{c_A}$$

$$\frac{c_{AT}}{c_A} = fraction\ bound = \frac{1}{2c_A} * \left(c_T + c_A + K_d - \sqrt{(c_T + c_A + K_d)^2 - 4c_T c_A}\right)$$

F_u	fluorescence in unbound state
F_b	fluorescence in bound state
K_d	dissociation constant, to be determined
c_{AT}	concentration of formed complex
c_A	constant concentration of molecule A (fluorescent), known
c_T	concentration of molecule T in serial dilution

Supplementary figure 3. Overexpression of PRINS regulates the expression and secretion of CCL-5. In parallel to priming with 5 ng/ml TNF-α and IFN-γ, NHEKs were transfected with a pcDNA3.1(+) vector containing the PRINS cDNA; an empty pcDNA3.1(+) vector was used as control. After 24 hours, cells were transfected with poly(dA:dT). RNA expression of CCL-5 was detected by real-time RT-PCR, secretion of CCL-5was measured by ELISA from cell supernatants. Data are presented as mean ± SE of four independent experiments. * p≤0.05; ** p≤0.01.

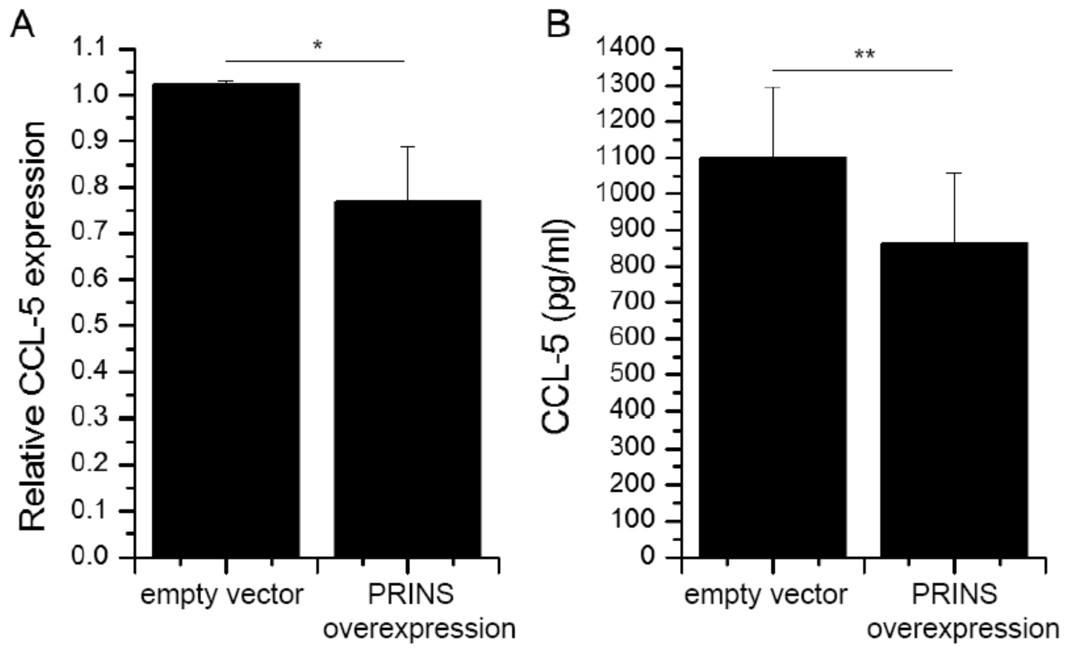

Supplement: Supplementary file 1 [file data_sheet_1.pdf]
